# A Cross-Subject Band-Power Complexity Metric for Detecting Mental Fatigue Through EEG

**DOI:** 10.3390/brainsci16020199

**Published:** 2026-02-07

**Authors:** Ang Li, Zhenyu Wang, Tianheng Xu, Ting Zhou, Xi Zhao, Honglin Hu, Marc M. Van Hulle

**Affiliations:** 1 Shanghai Advanced Research Institute, Chinese Academy of Sciences, Shanghai 201210, China; lia@sari.ac.cn (A.L.);; 2 University of Chinese Academy of Sciences, Beijing 100049, China; 3 Laboratory for Neuro- & Psychophysiology, Department of Neurosciences, KU Leuven—University of Leuven, B-3000 Leuven, Belgium; 4 School of Microelectronics, Shanghai University, Shanghai 200444, China

**Keywords:** EEG, electroencephalography, mental fatigue, cross-subject fatigue detection, N-Back task, driving fatigue

## Abstract

**Highlights:**

**What are the main findings?**

- The proposed Short-Term Second-Order Differential Entropy (ST-SODE) can capture fatigue from short-term band-power dynamics.
- ST-SODE improves the robustness of cross-domain EEG fatigue detection.

**What are the implications of the main findings?**

- ST-SODE reduces calibration burden for real-world fatigue monitoring.
- ST-SODE enables lightweight cross-subject deployment.

**Abstract:**

**Background/Objectives:** Electroencephalography (EEG) is a promising modality for fatigue detection because it directly reflects neural states; however, it is hindered by the need for subject-specific calibration and its reliance on unstable labeling. Moreover, classical EEG features are sensitive to intrinsic brain rhythm variations, causing pronounced domain shifts that degrade performance across sessions and subjects. **Methods:** Motivated by the biological fatigue rebound mechanism, we propose a robust cross-subject metric which we name Short-Term Second-Order Differential Entropy (ST-SODE). ST-SODE effectively suppresses the interference of background brain rhythms, enhancing robustness to cross-domain drift; consequently, its one-dimensional output can provide an indication of fatigue states without additional model training. **Results:** ST-SODE is validated on the public driving fatigue regression dataset SEED-VIG and on a private Vigilance classification dataset based on the N-Back task. ST-SODE achieves a correlation coefficient of 0.56 on SEED-VIG dataset (vs. 0.4 for differential entropy, DE) and a binary classification accuracy of 93.75% on the Vigilance dataset, outperforming other EEG-based fatigue metrics. **Conclusions:** ST-SODE offers a reliable solution for deployment in fields such as driving, manufacturing, and healthcare, where it could reduce safety incidents caused by fatigue.

## 1. Introduction

Statistics from the World Health Organization (WHO) show that 1.19 million people die from road traffic accidents every year [1]. Fatigue is one of the main culprits among the factors causing traffic accidents, predominantly in terms of rear-end car accidents; as such, fatigue is responsible for both human casualties and economic burden to society [2,3]. Fatigue reduces a driver’s vigilance, affecting the normal execution of driving tasks such as attention level, braking decisions, and reaction times [4,5]. Therefore, how to monitor and reduce driving fatigue has been a focus of research on traffic safety.

From the perspective of fatigue detection, three methodologies are used: physical feature identification [6], vehicle trajectory assessment [7], and biological signal assessment [8,9,10]. Biological signal assessment methods, among which Electroencephalography (EEG) stands out prominently, offer an effective and intuitive means of gauging a driver’s mental state. Consequently, signals such as EEG are frequently used to assess driver fatigue [11,12,13].

However, EEG signals are typically noisy and vary among subjects, leading researchers to develop a number of decoding methods. These methods primarily belong to two categories: the first involves traditional feature construction, in which time and frequency domain features such as Differential Entropy (DE) [14] and Power Spectral Density (PSD) [15,16] are constructed from EEG signals and supplied to machine learning classifiers; the second involves neural networks such as CNNs and RNNs [17,18,19,20], which can directly learn hidden features related to fatigue.

Although researchers have proposed various fatigue detection methods based on machine learning and deep learning, insufficient attention has been paid to the cross-session and cross-subject performance of these methods, which can lead to overfitting in practical applications because of lack of training data.

The brain exhibits rhythmic phenomena in which the power across different frequency bands of the brain varies over time and between subjects [21,22]. Classical fatigue features such as DE features use the magnitude of frequency-band power as an indicator of fatigue intensity; as such, they are susceptible to intrinsic fluctuations in brain rhythms, resulting in feature distribution shifts across subjects and across time periods. Deep learning models impose stricter requirements on feature distribution consistency, but these models typically need data recollection and model retraining for cross-subject and cross-session applications, which is a highly time-consuming process.

Therefore, this work aims to find a more robust metric to quantify mental fatigue. Fatigue is a protective response of the brain to prolonged high workloads. In realistic scenarios, the subject needs to maintain vigilance while driving or working. Through long-term experiments, we found that normally, when subjects feel tired, if they allow fatigue to develop, then they naturally fall into sleep. This is reflected by changes in the magnitude of the band’s power within the DE feature description [14]. When subjects fight against fatigue, such as during driving or working, this is manifested by fluctuation in the EEG frequency band power, known as the fatigue rebound effect [8,23,24]. In other words, as mental fatigue develops, EEG-band power does not exhibit a monotonic drift from vigilance to fatigue; instead, it typically progresses from a relatively stable alert state to a transient oscillatory phase, subsequently settling into a new stable state. Focusing on the fluctuations instead of on changes in the absolute magnitude helps to reduce the confounding effects of baseline cross-subject and cross-session variations.

Here, we use these fluctuations over a certain time window to describe the subject’s fatigue state during that period and propose the Short-Term Second-Order Differential Entropy (ST-SODE) metric to describe human fatigue. Because ST-SODE depends on fluctuation patterns rather than the absolute band-power magnitude, it is less sensitive to baseline shifts and scale differences across subjects and sessions, allowing for improved robustness under cross-subject and cross-session variability. We validated ST-SODE on a public fatigue dataset and a private binary vigilance–fatigue dataset. The results show that ST-SODE consistently outperforms existing metrics on both regression and cross-subject classification tasks. The contributions of this study are as follows:

- This study proposes using the fatigue rebound effect to assess fatigue levels, then introduces ST-SODE to characterize this phenomenon.
- ST-SODE describes the fluctuation of the short-term frequency-band power of the EEG signal without training; the one-dimensional output can directly represent fatigue states with cross-subject and cross-session robustness.
- We provide theoretical evidence that short-time DE features computed from long EEG sequences approximately follow a Gaussian distribution, which supports the statistical modeling assumptions of ST-SODE.
- This study includes a fatigue experiment based on the N-Back task for classification purposes. The proposed method was validated on both the N-Back dataset and the public SEED-VIG dataset. The results show that ST-SODE outperforms DE and PSD features in cross-subject scenarios using the Leave-One-Subject-Out (LOSO) strategy.

The rest of this paper is organized as follows: Section 2 introduces related works; Section 3 describes the proposed method; Section 4 introduces the datasets and experiment; Section 5 and Section 6 respectively present and discuss the results; finally, we conclude the paper in Section 7.

## 2. Related Works

An EEG-based fatigue detection system typically comprises four main components: signal acquisition, preprocessing, decoding, and postprocessing. Signal acquisition involves using an EEG cap to record the EEG signals associated with fatigue and vigilance states. Signal decoding forms the system’s core and includes two critical stages: feature construction or extraction, and decoding. Feature construction or extraction aims to derive key features that facilitate effective classification, employing methods such as the wavelet transform, Differential Entropy (DE) [25], Power Spectral Density (PSD) [16,26], etc. Extracted features such as entropy and time–frequency domain characteristics are utilized to differentiate between fatigue and vigilance states. Finally, features are decoded by training a classifier or regressor using machine learning methods. The results also require postprocessing (outlier detection, etc.) before application.

### 2.1. Feature Extraction

Methods for extracting EEG fatigue features primarily include time–frequency domain features and nonlinear features. Cui et al. [27] utilized the Fast Fourier Transform (FFT) to extract frequency-based features, integrating these with the Feature Weighted Episodic Training (FWET) classifier to detect fatigue. Zhang et al. [28] applied the Discrete Wavelet Transform (DWT) to extract entropy-based features, including the Wavelet Entropy (WE), Peak-to-Peak Approximate Entropy (PPApEn), and Peak-to-Peak Sample Entropy (PP-SampEn) to classify four driving fatigue stages. Duan et al. [25] were the first to employ DE features for driving fatigue detection. As a benchmark feature provided by the SEED-VIG dataset, DE has subsequently been widely cited and utilized in numerous studies [29,30]. When combining various features that have been proposed for EEG-based fatigue detection, the resulting high-dimensional feature space calls for dimensionality reduction or a feature selection process prior to or as part of the training process of the classifier or regressor. However, dimensionality reduction requires extensive labeled data, and challenges generalization to new subjects due to the cross-subject and cross-session variability of EEG signals.

### 2.2. Traditional Machine Learning Algorithms Applied in Classification

The classical machine learning methods of Support Vector Machine (SVM) and Support Vector Regression (SVR) for regression problems are extensively utilized in EEG-based fatigue detection. Shen et al. [31] proposed a probabilistic multiclass SVM detector for automatically classifying mental fatigue levels from EEG data. Li et al. [32] introduced the Auto-Correlation Function-based Sparse Support Matrix Machine (ACF-SSMM) algorithm, which incorporates location information into EEG signals and employs sparsity to reduce redundant features, resulting in enhanced fatigue detection performance. Wang et al. [33] extracted four graph-related features using Partial Directed Coherence (PDC)-based brain functional network construction. They utilized SVM for offline classification, achieving an accuracy of 87.16% for binary classification in a simulated driving experiment. For the SEED-VIG dataset labeled with continuous values, Zheng et al. [34] applied Differential Entropy (DE) features and Support Vector Regression (SVR) for regression-based prediction. In addition to SVM-based methods, other machine learning classifiers have also been applied to EEG-based fatigue detection, such as K-Nearest Neighbor (KNN) [35], Bayesian Neural Network (BNN) [36], and Feature Weighted Episodic Training (FWET) [27].

### 2.3. Deep Learning Models

Compared to traditional machine learning algorithms, deep learning methods require larger training datasets but offer significantly better prediction performance on test data within the same subject. Convolutional Neural Networks (CNN) are widely employed in image recognition and classification [17,18,37]. They possess the ability to automatically and adaptively learn the hierarchical feature space at each level, which effectively reduces the number of learned parameters [17]. Recent studies have highlighted the strong feature extraction capabilities of CNNs in EEG analysis. For instance, Wu et al. [18] proposed a deep sparse contractive autoencoder network combined with Finite Impulse Response (FIR) filters to extract local features and predict fatigue status, achieving 94.58% classification accuracy on a three-class private flight simulation fatigue dataset. Similarly, Hu et al. [19] introduced the Spatio-Temporal Fusion Network with Brain Region Partitioning Strategy (STFN-BRPS), a multi-branch deep learning network designed to enhance the accuracy and robustness of EEG-based driver fatigue detection. The model incorporates a recurrent multiscale convolution module for temporal features, a dynamic graph convolution module for spatial features, and a channel attention-based feature fusion module. This approach demonstrated superior performance compared to conventional methods on real-world driving tests.

While deep learning enhances feature extraction performance for specific domains, it is susceptible to overfitting [38]. EEG data exhibit significant variability across subjects [39,40], which means that new data must be collected from each subject in order to retrain the model or recalibrate the original model. This requirement poses a major challenge to the practicality of EEG-based fatigue detection methods.

## 3. Methods

### 3.1. EEG Frequency Bands

An Electroencephalograph (EEG) is a biosignal representing changes in surface potentials on the scalp, which reflect the electrophysiological activity of neural cells in the brain. EEG features are generally defined by their distinct frequency ranges and spatial patterns. Functionally, EEG frequency domain features are categorized into the five frequency bands shown in Table 1. Notably, the alpha and theta bands are strongly associated with fatigue [41,42,43,44]. Specifically, when subjects are mentally fatigued, their theta-band power will increase significantly. Their alpha-band power also increases; however some studies have observed weakening of the alpha band due to engagement in task-related areas [45]. The beta-band power may also weaken; however, the specific performance varies greatly between subjects.

To provide a more stable representation of fatigue, the frequency-band ratios θ/β, α/β, (α+θ)/β and (α+θ)/(α+β) are commonly used. This approach helps to mitigate baseline shifts that often occur when relying solely on a single frequency band over extended periods. However, the power within EEG frequency bands is sensitive to both long-term shifts and transient fluctuations caused by intrinsic physiological states or external environmental factors. These short-term variations can lead to instability in features from combined frequency bands, posing challenges for accurate fatigue prediction. Therefore, predicting the fatigue state of a subject solely based on the increase or decrease in frequency-band power is unreliable. In practical applications, frequency-band power features are extracted first, then machine learning methods are applied to learn their characteristics.

### 3.2. Short-Term Second-Order Differential Entropy

**Figure 1:**
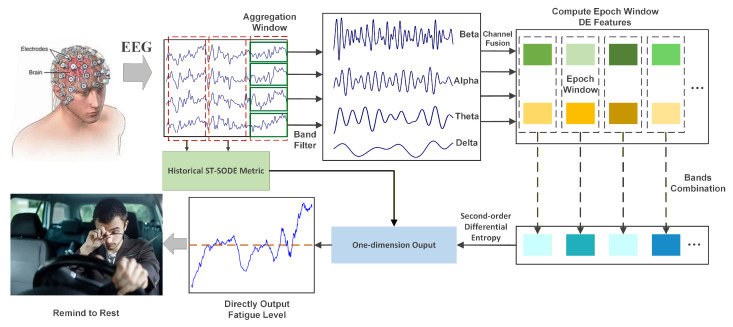
Scheme of the proposed ST-SODE fatigue estimation framework; the multichannel scalp EEG is segmented into aggregation windows, then band-pass filtered into different epoch-level band powers. The extracted DE features and the equivalent second-order differential entropy of the aggregation windows are used to describe the intensity of the fatigue rebound effect, which indicates the mental fatigue level.

The framework of the proposed Short-Term Second-Order Differential Entropy (ST-SODE) method is illustrated in Figure 1. To quantify fatigue levels using EEG signals, we employ a dual-windowing strategy comprising the epoch window ($T_{e}$) and the aggregation window ($T_{a}$). The epoch window $T_{e}$ is a short-time window used to extract the instantaneous spectral features, while the aggregation window $T_{a}$ is a longer sliding window designed to capture the temporal variability of these features. Let $N_{e}$ denote the number of epoch windows contained within one aggregation window, $f_{s}$ the sampling rate, and *s* the fixed step size between adjacent epoch windows.

For each epoch window $T_{e}$, the power spectrum is computed using Welch’s power spectral density estimate [46]:

(1) $$P_{T_{e}}\left(f\right)=\frac{1}{N}{\left|\sum_{n=0}^{N-1}w\left(n\right)\cdot x\left(n\right)e^{-j2\pi fn/f_{s}}\right|}^{2}$$

where w(n) is the Hamming window, represented as

(2) $$\begin{array}{c} w\left(n\right)=0.54-0.46\cos\left(\frac{2\pi n}{N-1}\right),\\ \;n=0,1,2,\ldots ,N-1. \end{array}$$

Then, the band power $E_{i}$ is calculated in four frequency bands (delta: 0.5–4 Hz theta: 4–8 Hz, alpha: 8–12 Hz, beta: 13–30 Hz). The Differential Entropy (DE) was initially defined as

(3) $$\begin{array}{cc} h(X)\; & =-\int_{-\infty }^{\infty }f_{X}\left(x\right)\;\log f_{X}\left(x\right)\;dx\\ \; & =-\int_{-\infty }^{\infty }\frac{1}{\sqrt{2\pi \sigma^{2}}}\exp\;\left(-\frac{{\left(x-\mu \right)}^{2}}{2\sigma^{2}}\right)\log\;\left[\frac{1}{\sqrt{2\pi \sigma^{2}}}\exp\;\left(-\frac{{\left(x-\mu \right)}^{2}}{2\sigma^{2}}\right)\right]dx\\ \; & =\frac{1}{2}\log\;\left(2\pi e\sigma^{2}\right), \end{array}$$

with the time series *X* assumed to follow a Gaussian distribution $N(\mu ,\sigma^{2})$. It has since been proven that for a fixed-length EEG sequence, the DE is equivalent to the logarithm of the energy spectrum or the signal variance in a certain frequency band [14].

Subsequently, based on Section 3.1, the logarithm of the energy spectrum $E_{i}$ features is calculated. To capture the global state, the features are pooled by averaging across all channels and combined through frequency band i∈{δ,θ,α,β} to obtain the final DE feature $D_{\phi }$ for each epoch window:

(4) $$D_{\phi }=\phi \left\{DE_{\delta },DE_{\theta },DE_{\alpha },DE_{\beta }\right\},$$

where ϕ represents the different band-power combinations discussed in Section 3.1.

Next, we prove that the distribution of DE features of windows from long-length EEG signals approximates a similar Gaussian distribution. Let signal segment *X* of length *N* follow a stationary Gaussian distribution, denoted as $X∼N(\mu ,\sigma^{2})$. The estimator for the Differential Entropy (DE), denoted as $\hat{h}$, is provided by

(5) $$\hat{h}=\frac{1}{2}\ln\left(2\pi eS^{2}\right),$$

where $S^{2}$ represents the sample variance of the segment. According to the Central Limit Theorem [47], as the sample size N→∞, the sample variance $S^{2}$ exhibits asymptotic normality:

(6) $$\sqrt{N}\left(S^{2}-\sigma^{2}\right)\overset{d}{\to }N\left(0,2\sigma^{4}\right).$$

This implies that for large *N*, $S^{2}$ is approximately distributed as $N(\sigma^{2},\frac{2\sigma^{4}}{N})$.

To derive the distribution of $\hat{h}$, we apply the Delta method [48]. Let $g\left(u\right)=\frac{1}{2}\ln\left(2\pi eu\right)$, such that $\hat{h}=g\left(S^{2}\right)$. The first-order Taylor expansion of $g(S^{2})$ around the true variance $\sigma^{2}$ is

(7) $$\hat{h}\approx g\left(\sigma^{2}\right)+g^{\prime }\left(\sigma^{2}\right)\left(S^{2}-\sigma^{2}\right).$$

The derivative of the function is $g^{\prime }\left(u\right)=\frac{1}{2u}$. Evaluated at $\sigma^{2}$, we have $g^{\prime }\left(\sigma^{2}\right)=\frac{1}{2\sigma^{2}}$.

Because a linear transformation of a Gaussian random variable remains Gaussian, $\hat{h}$ follows a normal distribution asymptotically. The variance of $\hat{h}$ is derived as follows:

(8) $$\begin{array}{cc} \mathrm{Var}(\hat{h}) & \approx {\left[g^{\prime }\left(\sigma^{2}\right)\right]}^{2}\cdot \mathrm{Var}\left(S^{2}\right)\\ \; & ={\left(\frac{1}{2\sigma^{2}}\right)}^{2}\cdot \frac{2\sigma^{4}}{N}\\ \; & =\frac{1}{4\sigma^{4}}\cdot \frac{2\sigma^{4}}{N}=\frac{1}{2N}. \end{array}$$

Consequently, as N→∞, the segmented DE values follow a similar Gaussian distribution:

(9) $$\hat{h}∼N\left(\mu_{real},\frac{1}{2N}\right).$$

Based on this proof, over the aggregation window $T_{a}$ containing $N_{e}$ epoch windows, we calculate the ST-SODE feature as the variance of the sequence $\left\{D_{\phi }\left(T_{e_{1}}\right),\ldots ,D_{\phi }\left(T_{e_{n}}\right)\right\}$. The ST-SODE is given by

(10) $$\begin{array}{c} S_{\Phi }\left(T_{a}\right)=\frac{1}{N_{e}-1}\sum_{i=1}^{N_{e}}{\left(D_{\Phi }\left(T_{e_{i}}\right)-{\bar{D}}_{\Phi }\left(T_{a}\right)\right)}^{2}, \end{array}$$

where ${\bar{D}}_{\Phi }\left(T_{a}\right)$ is the mean DE value over the aggregation window and $N_{e}$ is the number of epoch window in one aggregation window. It has been proven in [14] that the variance estimation of signal sequence is just its average energy; therefore, the above operation can be regarded as a simplified version of the DE computation. We do not further apply a logarithmic operation here, since the potential negative values are undesirable for directly outputting the fatigue index. However, due to the monotonicity of the logarithm, its variation trend remains strictly consistent with that of the standard DE. A larger ST-SODE value indicates greater fluctuation and instability in the relative activity between the two frequency bands.

## 4. Datasets and Experiment Setup

In this study, we utilized two EEG-based fatigue datasets. The first public dataset, SEED-VIG, was collected by Shanghai Jiao Tong University and contains EEG signals recorded during simulated driving tasks. Eye-tracking devices were employed to monitor blink and closure, which served as the basis for fatigue regression labeling. The second dataset is a private dataset using the N-Back task to induce fatigue in subjects. In a typical N-Back paradigm, subjects continuously respond to a stream of stimuli by indicating whether the current stimulus matches the one presented N steps earlier, thereby demanding working memory and sustained attention. The N-Back task was initially introduced by Kirchner in [49] to impose varying cognitive workloads. Subsequently, Tanaka et al. [50] and Pergher et al. [51] demonstrated that the N-Back task can effectively induce fatigue when its design closely mirrors that of the control 1-Back task. The 1-Back task can induce fatigue, but less than the 2-Back task because of the higher task difficulty. Other fatigue classification studies have also employed N-Back experiments to induce varying degrees of fatigue [52,53]. Detailed descriptions of the experiment setup and data formats for both datasets are provided in the following subsections.

### 4.1. SEED-VIG

The first dataset was compiled and released by a research team at Shanghai Jiao Tong University [34]. They developed a simulated driving system to collect EEG data. The system was composed of a large LCD screen and a real vehicle without engine and other components. The vehicle was modified so the participants could operate the vehicle through the steering wheel and gas pedal using a screen in front of them. To induce fatigue, the LCD screen displayed a primarily straight and monotonous four-lane highway scene.

A total of 23 volunteers with an average age of 23.3 years participated in the fatigue driving experiments. To facilitate the induction of driving fatigue, subjects were required to participate in the experiment in the afternoon or late at night. The experiment lasted about 2 h, during which data were recorded. Both EEG and forehead EOG signals were recorded during the fatigue driving experiment; however, only EEG data are analyzed in this paper. EEG signals were recorded from eleven channels at the posterior site and six channels at the temporal site, following the international 10–20 electrode system. The sampling rate was 200 Hz. An annotation, called the PERCLOS measure, was adopted to quantify subjects’ fatigue. PERCLOS indicates the percentage of eye blink and closure, with the PERCLOS value between 0 (vigilance) and 1 (fatigue).

### 4.2. Vigilance Dataset

#### 4.2.1. Experimental Protocol

The second dataset consists of a binary vigilance–fatigue dataset collected from eight subjects. To induce fatigue, we designed an experiment based on cognitive load experiments in psychology. In the N-Back task, participants are presented with a sequence of stimuli, which can be either visual or auditory, and must identify whether the current stimulus matches the one presented “N” steps earlier in the sequence. The value of “N” can be varied to adjust the difficulty of the task, with larger values requiring participants to hold more items in memory and track them over longer intervals.

**Figure 2:**
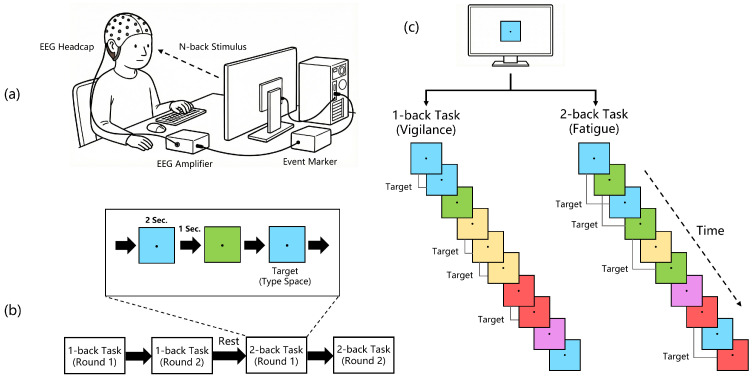
The N-Back experiment used to construct the Vigilance dataset. (**a**) Experimental setup: The experimental equipment included a NeuroScan SynAmps2 amplifier system, an electrode cap, and a computer with a monitor. (**b**) Experimental procedure: Two rounds of 1-Back tasks and two rounds of 2-Back tasks were conducted, with a 10-min rest period between 1-Back and 2-Back tasks. The 1-Back task was used to represent a vigilant working state, while the 2-Back task was used to induce the fatigued working state. (**c**) Explanation of N-Back experiments: Participants were told to press the spacebar when the current color block matched the previous one (1-Back) or the one before that (2-Back). The 2-Back task required sustained recall of historical color blocks within a short timeframe, resulting in significantly higher workload than 1-Back and rapidly inducing fatigue.

In this study, we used different colors as stimuli and carried out two tasks for each subject, as illustrated in Figure 2. The 1-Back task was used to represent a relative vigilant working state, while the 2-Back task was used to induce the fatigue working state. To minimize interference between the two tasks, each subject first completed the 1-Back task; after a 10-min rest, the same subject then performed the 2-Back task. For the 1-Back task, the monitor displayed changing color blocks, each lasting 2 s with an interval of 1 s. The subject was instructed to concentrate on the screen and press the space bar when two consecutive colors were the same. For the 2-Back task, the subject was instructed to press the space bar when the current color was the same as two steps prior. Both tasks consisted of two rounds of 5 min each, with no interval between them.

Eight healthy subjects were recruited, seven males and one female (age: 23.75 ± 2.11 years, mean ± standard deviation). All subjects reported normal or corrected-to normal vision, with no history of substance addiction or mental disorders. The subjects were required to obtain a full night (>7 h) sleep before the day of the experiment. On the day of the experiment, they were required to avoid consuming caffeine or alcohol. The experiment was conducted in an electromagnetically shielded room. Before the start of the experiment, the subjects were trained to familiarize themselves with the N-Back task and to understand the necessity of completing the tasks as accurately as possible even when feeling tired. The study complied with the principles of the 2024 Declaration of Helsinki and was approved by the Research Ethics Committee of Shanghai University (no. ECSHU 2024-072). All subjects signed informed consent forms.

#### 4.2.2. EEG Data Acquisition

**Figure 3:**
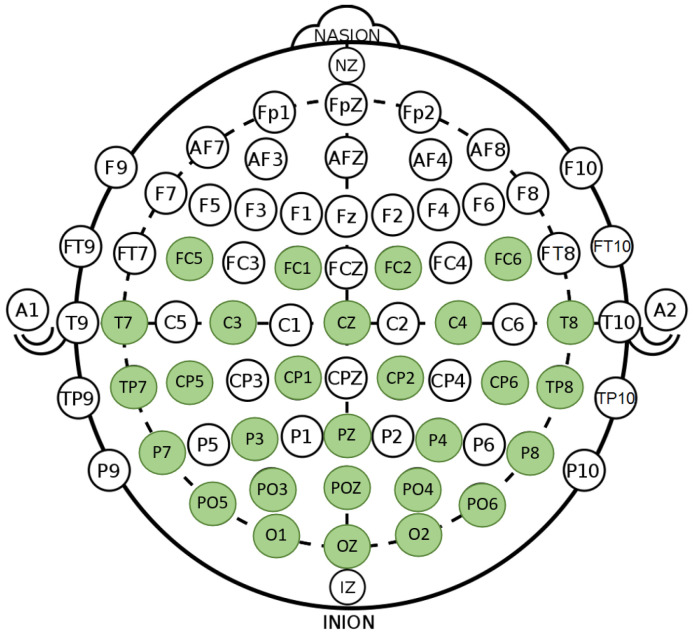
Position of the 28 electrodes during construction of the Vigilance dataset. Electrodes labeled in green were used to model fatigue. The reference electrode (default REF) was located between CZ and CPZ.

Brain activity was recorded using EEG recording equipment (Compumedics Neuroscan, Charlotte, NC, USA) with 28 wet electrodes using the 10–20 system (without forehead electrodes, according to SEED-VIG). The selection and position of the channels are shown in Figure 3. The sampling rate was 1000 Hz. The impedances of all EEG channels were kept below 10 kΩ.

### 4.3. Experiment Setup

#### 4.3.1. Effectiveness Experiment on SEED-VIG

The label of SEED-VIG is a floating value from 0 to 1; thus, we use the prediction curves and correlation analysis to demonstrate the performance of ST-SODE. The SEED-VIG dataset contains three parts: EEG (temporal lobe and posterior lobe), Forehead EEG, and EOG. Only the EEG data were utilized in this experiment, with the Forehead EEG and EOG data left out. Forehead electrodes can cause eye movement artifacts; after these data were removed, the raw EEG data were processed with a fourth-order Butterworth band-pass filter between 0.5 and 45 Hz to reduce artifacts and noise, referencing [34]. The filtering was completed before epoch window segmentation to reduce the edge effect. The epoch window size was set to 0.5 s and the aggregation window size to 8 s. The epoch window strategy was non-overlapping, with the step size the same as the epoch window length. After ST-SODE, the result was min–max normalized, then displayed in the same figure with PERCLOS labels.

#### 4.3.2. Vigilance Dataset

The Vigilance dataset involves two tasks with different levels of workload, namely, the 1-Back task and 2-Back task. Each task consisted of two rounds, each of which began with the cue “Round 1/2 begins”. We selected the first round of the 1-Back task as the vigilant case and the second round of the 2-Back task as the fatigue case. The preprocessing was the same as SEED-VIG. The epoch window size was set at 0.5 s and the aggregation window size at 5, 10, and 20 s to assess the effect on the results. Each subject had 120, 60, and 30 trials for 5 s, 10 s, and 20 s, with fatigue and vigilance being equally divided. To classify fatigue without training on the test subject, we calculated the average value of the obtained segmented ST-SODE features from the other seven subjects, excepting the test subject. The training data length was [840/420/210, 5/10/20 * 1000 (Hz)] (trials, data points). Features from the test subject that were above the average value were predicted as fatigue, and vice versa as vigilance. The Differential Entropy (DE) and Power Spectral Density (PSD) were employed as a comparison. DE features were processed consistently with ST-SODE, and a Support Vector Machine with a Gaussian kernel was used to classify PSD features using the LOSO strategy, with the data of the other seven subjects excepting the test subject used for training. To compare the performance of ST-SODE with supervised subject-specific methods, we also conducted within-subject 5-fold cross-validation experiments using the DE and PSD features with supervised SVM classifiers.

In addition, we calculated the average ST-SODE results of fatigue and vigilance trials for each subject.

## 5. Results

### 5.1. Performance on SEED-VIG

**Figure 4:**
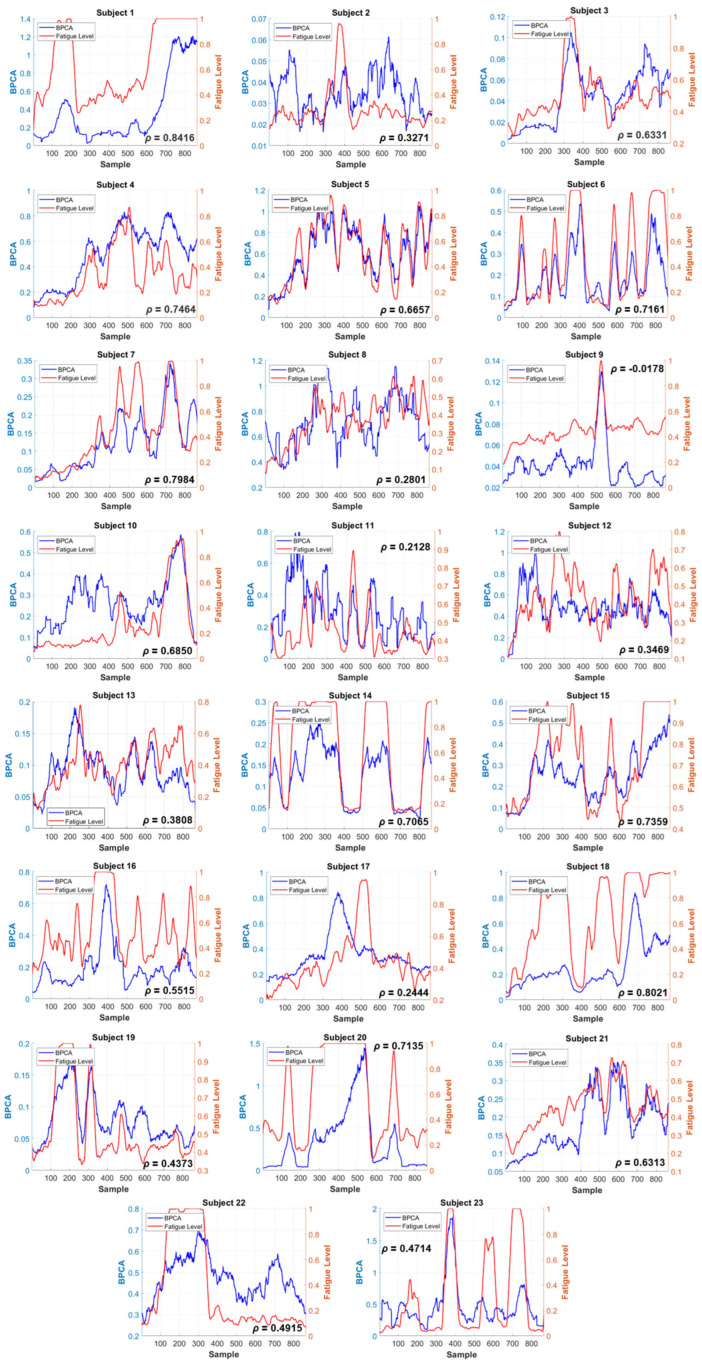
Visualization results of ST-SODE on the SEED-VIG dataset. The red line is the fatigue Level given by PERCLOS, with a high value indicating fatigue, while the blue line is the predicted result. Each figure is labeled with the correlation coefficient ρ. The sampling length is 8 s. Because the PERCLOS label is a discrete value derived by the eye tracker in another modality and the prediction is made without training or any use of label information, the two curves do not align precisely. Nonetheless, it is still possible to observe temporal synchronization by examining the rising and falling edges of the two curves.

A comparison of the PERCLOS labels and ST-SODE results among all subjects is depicted in Figure 4. ST-SODE (blue line) exhibits varying degrees of correlation with PERCLOS (red line), which are collected in Table 2. The performance is divided into three categories, as discussed below:

- Most experiments show significant correlation between ST-SODE and PERCLOS changes: S01, S03, S04, S05, S06, S07, S10, S14, S15, S17, S18, S20, and S21. All their correlation coefficients are above average 0.56. Their peaks and troughs along the rising and falling edges remain largely coincident. Because ST-SODE and PERCLOS are both complete data modalities, the heights of the two curves may differ.
- The second category consists of correlation coefficients between 0.2 to 0.56 but which still exhibit strong correlation in the image. This is specifically manifested as follows: when PERCLOS shows a peak in a short period of time, ST-SODE also demonstrates a similarly temporally coincident fluctuation. This category contains S11, S12, S13, S16, S19, S22, S23.
- The third category comprises subjects with a correlation coefficient below 0.2. This group includes S02, S08, and S09. For S09, it can be observed that the two curves coincide almost exactly when there is a crest.

At the same time, we also calculated the performance of PSD and DE, which are widely used as features in fatigue prediction. In order to show the result without training, the processing method of PSD and DE was kept consistent with ST-SODE. The feature average of all channels was calculated to obtain a one-dimensional time series, then the correlation coefficient was calculated with the label. The results are shown in Table 2.

According to Table 2, when the PSD and DE features are not trained using classifiers with labels, their correlation with the labels is lower than ST-SODE. Among the four different frequency band combinations, the highest PSD is only 0.0325, the DE is 0.4001, and the chance level of the random signal between 0 and 1 is 0.005. The untrained ST-SODE method can achieve an average correlation coefficient of 0.56, significantly higher than DE or PSD (** *p* < 0.01 and *** *p* < 0.001, Wilcoxon signed-rank), which proves the effectiveness of ST-SODE in predicting fatigue without training.

### 5.2. Performance on Vigilance Dataset

**Figure 5:**
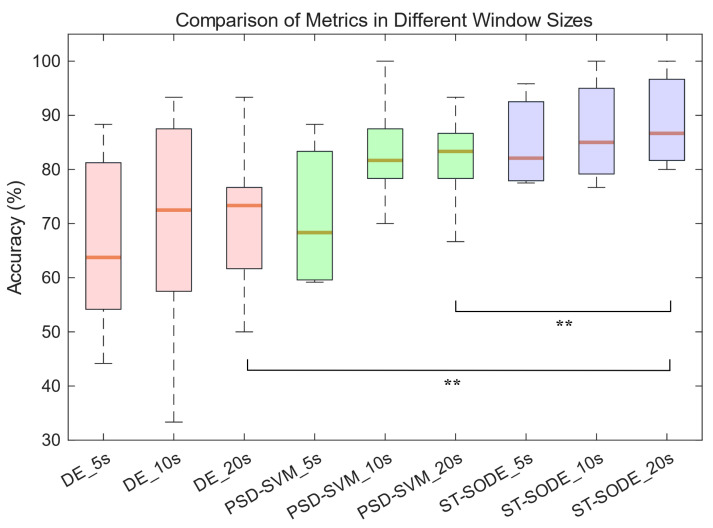
Comparison of DE, PSD-SVM, and ST-SODE on the Vigilance dataset. Three window settings (5 s, 10 s, and 20 s) are applied. PSD-SVM uses cross-subject setting (data from the seven subjects other than the test subject were used for training) in order to remain consistent with the untrained ST-SODE method. Here, ** indicates *p* < 0.01 on the Wilcoxon signed-rank test.

The classification results of ST-SODE and the two compared methods (DE and PSD) are shown in Table 3 and Figure 5. Each method employs three different aggregation window lengths (5S, 10S, 20S).

It can be observed that the mean accuracy of ST-SODE across all three window lengths significantly exceeds those of the DE and PSD-SVM methods. ST-SODE achieves the highest average accuracy (93.75%) at a window length of 20 s, which is 6.46% higher than at 5 s and 1.46% higher than at 10 s. However, while increasing the sampling window length improves accuracy, it also leads to a reduction in system response speed. For the DE and PSD methods, accuracy significantly improves as the window length increases from 5 s to 10 s, with gains of 3.86% and 11.88%, respectively; however, when the sampling window length is further extended from 10 seconds to 20 s, DE and PSD show minimal accuracy gains or even slight declines, with changes of 0.62% and −1.05%, respectively. We believe that this is because keeping the total data length fixed while increasing the size of the sampling window results in a reduced number of training samples.

To quantify the magnitude of performance differences beyond statistical significance, we additionally report the rank-biserial correlation *r* as an effect-size measure for the paired Wilcoxon signed-rank test. The value of *r* ranges from [−1,1], with r>0 indicating that ST-SODE achieves higher accuracy than the comparator and larger |r| implying a stronger and more consistent advantage across subjects. Based on the subject-wise paired accuracy results in Table 3, ST-SODE exhibits positive effect sizes over DE, with r=0.72/0.50/0.69 for window lengths of 5 s/10 s/20 s. Compared to PSD-SVM, the effect sizes are r=0.67/0.29/0.52 for 5 s/10 s/20 s, respectively. Overall, these results suggest that ST-SODE provides a consistent improvement over both DE and PSD-SVM across subjects.

In Table 4, we report the performance comparison between the cross-subject ST-SODE method and DE/PSD features with supervised SVM classifiers. The comparison indicates that the within-subject supervised method DE-SVM achieves the highest accuracy (97.50–98.44%), representing an accuracy improvement of 3.75–11.15% at the 20 s and 5 s window settings compared to the cross-subject ST-SODE. This performance gap reflects the advantage of subject-specific calibration in capturing individual EEG characteristics. Therefore, the choice of whether to sacrifice accuracy in order to avoid subject-specific calibration represents a tradeoff that users must evaluate based on their practical requirements.

In Table 5, it can be observed that the average of ST-SODE on the vigilance task is significantly lower than on the fatigue task (** *p* < 0.01). For example, when using an aggregation window length of 5 s, the average of ST-SODE on the vigilance task data is 0.000077, while on the fatigue task data it reaches 0.064361. Although ST-SODE scores higher for some subjects on the vigilance task, such as Subject 5 with 0.000257, this is still much lower than the lowest score on the fatigue task (Subject 1 with 0.000893). Meanwhile, the smallest difference between vigilance and fatigue is also from Subject 1, with 0.000079 and 0.000893, respectively, while the largest difference is from Subject 5, with 0.000257 and 0.219803.

### 5.3. Effectiveness Analysis

#### 5.3.1. Performance When Using Other Band Power Ratios

ST-SODE uses α/β as the band-power combination due to its high performance compared to other combinations. We calculated the Spearman correlation between the predictions and PERCLOS on SEED-VIG using different band power ratios of θ/β, α/β, (α+θ)/β and (α+θ)/(α+β), with the results shown in Table 2. The chance level is calculated using the shuffled label and the results of α/β.

From Table 2, it can be seen that α/β achieves the highest correlation among the four methods. The results obtained from the other three combinations are poorly correlated with the PERCLOS labels.

#### 5.3.2. Feature Visualization

**Figure 6:**
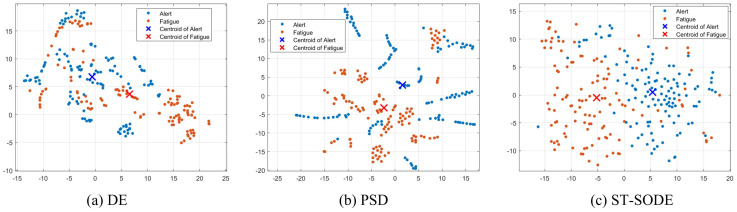
t-SNE visualization of DE, PSD, and ST-SODE features. All subject data from the Vigilance dataset are combined.

To validate the representation ability of the proposed method, we plot the t-SNE [54] transformed features in Figure 6.

The t-SNE is a nonlinear manifold learning technique that projects high-dimensional feature vectors into a low-dimensional space (typically 2D) for visualization by preserving local neighborhood relationships. Therefore, points that are closer together on the 2D map generally indicate more similar representations.

Specifically, we exhibit the DE, PSD, and ST-SODE features extracted from all subjects’ data in the Vigilance dataset. We used a learning rate of 500 and an iterative display frequency of 20 for the t-SNE calculation.

From the visualized features represented in the figure, it can be observed that the ST-SODE features are more class-discriminative than the other features. Additionally, the DE features are distributed in several regions which contain both vigilance and fatigue features. This issue illustrates why decoding methods are prone to significant deterioration on cross-subject tasks. Meanwhile, it is notable that some points of the PSD features are close to straight lines, which proves that features from the same subject in the same state are very similar compared to ST-SODE and DE while being very different from other subjects. This proves the difficulty of training a classifier that is universal for all subjects, as it is challenging to learn a hyperplane (or neural network) that can effectively distinguish the two types of states based on the confusing features.

## 6. Discussion

### 6.1. Topography of Channel-Level ST-SODE

**Figure 7:**
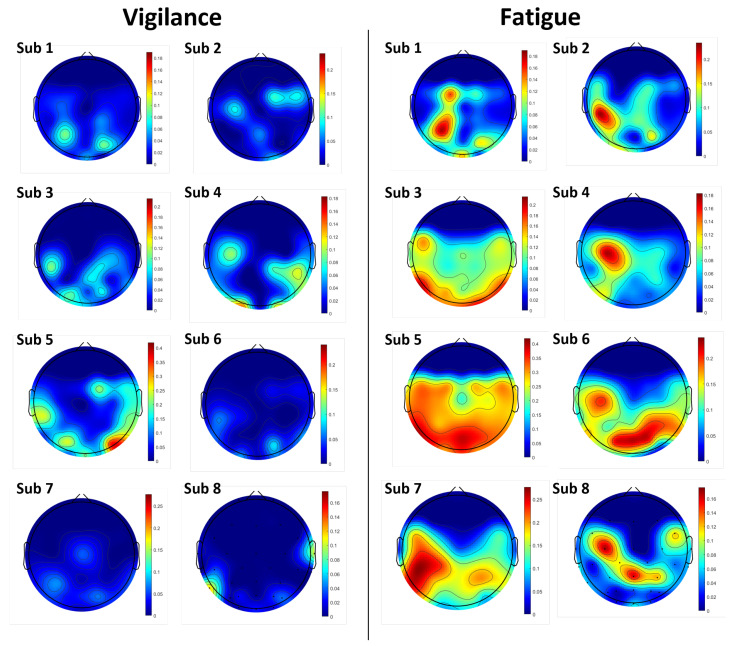
Channel-level ST-SODE brain topography. We calculated ST-SODE separately for each channel, with the aggregation window set to 5 s. The experiment did not include frontal electrodes. The color-bar range is fixed for each subject in terms of vigilance and fatigue; red indicates higher band-power fluctuations, signifying a more severe state of fatigue, while blue represents vigilance.

The standard ST-SODE does not focus on the features between channels, but instead averages the values of all channels. To explore the spatial variation of ST-SODE across the scalp, Figure 7 shows the channel-level ST-SODE topography.

Notably, the fatigue condition shows significant increases in ST-SODE values within particular cortical regions for most subjects. Specifically, Subjects 1, 2, 4, 7, and 8 exhibit high ST-SODE activity in the left temporal electrodes and left parietal electrodes, while Subjects 5 and 6 show stronger ST-SODE features in the occipital electrodes.

This suggests that it is difficult to use individual channels for ST-SODE detection. The spatial differences in performance during fatigue and non-fatigue are different across subjects; therefore, attempting to use all of the channels for ST-SODE analysis can enhance generalizability to all subjects. However, when considering realistic application scenarios such as driver fatigue detection, it may not be possible to provide electrode caps that cover the whole brain, as in laboratory scenarios. Consequently, employing localized portable electrode setups centered on the left posterior region would optimize detection efficacy within these practical constraints.

### 6.2. Importance of Cross-Subject Method

Previous studies have shown that neuronal activities exhibit variability in the frequency and time domains over the states of vigilance and fatigue [11,12]. This variability is mainly exhibited by changes in the frequency domain features. Recent neurophysiological studies have suggested that this phenomenon captured by ST-SODE is likely attributable to Locus Coeruleus (LC) oscillations [55,56]. During fatigue, LC neurons exhibit functional fatigue and infra-oscillations. These fluctuations in noradrenergic gain cause cortical networks to oscillate between low-excitability and high-excitability states, generating the high spectral variance that ST-SODE measures. This mechanism aligns with observations by Doran et al. [57], who noted unstable fluctuations in attentional states during sleep deprivation. Most existing studies focus on utilizing a classifier trained on a single subject. This provides stronger fitting ability, but at the same time constrains realistic applications due to the need for subject-specific training data. In real-world situations, especially driving fatigue detection, there is urgent need for a white-box algorithm with clear internal workings and decision-making logic.

ST-SODE metrics effectively meet this requirement. Our findings reveal significant power fluctuation on EEG bands when subjects are in a fatigued state. The proposed ST-SODE method is designed based on this phenomenon to be white-box and training-free, and as such can be used in realistic fatigue detection scenarios.

Compared to PSD and DE features, ST-SODE features are less susceptible to shifts in frequency-band power across session and subject. Under normal conditions, frequency-band power varies with circadian rhythms [58] and arousal levels [59], making it challenging to evaluate fatigue changes using absolute power values across different periods. In contrast, ST-SODE features are unaffected by absolute value changes, enabling consistent fatigue standards to be applied for monitoring drivers across various sessions.

It is important to clarify the definition of “calibration-free” in this context. Although ST-SODE does not require any subject-specific calibration (where a new subject must provide labels to train a personal model), it still requires preparation for the classification threshold. As demonstrated by the LOSO setting, the threshold is derived from existing subjects. Therefore, the system preparation phase requires a database of labeled subjects in order to establish the threshold before the system can be applied to new subjects.

### 6.3. Labels of Fatigue Experiment

One of the major challenges in fatigue detection is labeling. The fatigue state of a subject is highly random and unstable, and is also affected by various factors. Sleeping state, circadian rhythms, task difficulty, and physical differences can all affect the fatigue level of the subject [60,61]. Most fatigue monitoring methods use supervised learning methods that require labeled data to fit a classifier [25,27,28,29,62], in which case the accuracy of the labeling affects the performance of the model for fatigue prediction.

Previously, fatigue was usually inferred indirectly by analyzing subject metrics such as eye blinks [63], frequency of yawns [64], and task errors [65]. However, there are many other factors associated with these signs. A classifier obtained by training on indirect labels still predicts indirect labels, leaving the correlation between fatigue level and indirect labels in need of further validation.

Our Vigilance dataset used cognitive load to induce the fatigue state. It was designed to produce a similar state to driving or performing other long-time tasks by means of a high-difficulty repetitive task over a short period of time, while utilizing a low-difficulty task to control for variables. We designed the metrics with reference to the properties of the brain when fatigued in order to avoid introducing other disturbances using supervised learning.

The test results on the Vigilance dataset demonstrate that ST-SODE metrics can effectively distinguish between two different work states. The process is a result-oriented one, and we consider that fatigue can be effectively detected if the two states can be distinguished.

### 6.4. Limitations

Despite its promising results, this study still has limitations that need to be addressed in future work. First, while ST-SODE achieves promising performance on the SEED-VIG dataset, poor performance on some subjects indicates that this metric alone is currently insufficient for operational deployment in safety critical scenarios, and should ideally be integrated into multimodal and distributed monitoring systems [66] to ensure reliability. Second, the Vigilance dataset employed an N-Back task to induce fatigue through high cognitive load, which differs from the vigilance decline typical of real-world driving; consequently, the generalizability of these findings to real driving conditions requires further validation in future on-road experiments.

Third, the frequency-band ratio (α/β) utilized in ST-SODE was selected based on its best performance on the SEED-VIG dataset. While this combination demonstrated effectiveness for the Vigilance dataset, we acknowledge that this selection is empirical. Therefore, when applying ST-SODE to new scenarios, users would need to obtain labeled data from their domain in order to determine the optimal frequency-band combination.

Last, although proportional drift across bands offers a reasonable hypothesis for the effectiveness of ST-SODE in cross-subject scenarios, the mechanistic explanation remains empirically demonstrated but theoretically underspecified in the current study. Conclusive validation of these specific drift patterns requires further targeted research into the neurophysiological mechanisms.

## 7. Conclusions

This study has proposed a cross-subject EEG-based fatigue metric called ST-SODE which can be applied to scenarios such as driving, manufacturing, and healthcare that require low fatigue levels. ST-SODE utilizes the fatigue rebound effect to represent fatigue levels; this approach reports fatigue levels by calculating the fluctuation of short-window DE features, equivalent to the second-order differential entropy. We validated the effectiveness of ST-SODE on the public SEED-VIG dataset as well as on a private dataset based on the N-Back task. ST-SODE achieved a correlation coefficient of 0.56 on the SEED-VIG regression dataset and 93.75% binary classification accuracy on the Vigilance dataset, outperforming other metrics under the same LOSO settings. ST-SODE can be deployed in existing environments while requiring no subject-specific calibration, thereby addressing the limitations of supervised fatigue detection methods in real-world applications.

## Figures and Tables

**Table 1:** Frequency bands of EEG signals.

Number	Frequency Band	Frequency Range/Hz	Amplitude/μV
1	Delta (δ)	0.5–4	10–20
2	Theta (θ)	4–8	20–40
3	Alpha (α)	8–12	10–100
4	Beta (β)	13–30	5–30
5	Gamma (γ)	30–100	≈40

**Table 2:** Correlation between prediction and labels using different frequency-band settings without training.

Method	DE				PSD				ST-SODE				
**Sub**	θ/β	α/β	(α+θ)/β	(α+θ) /(α+β)	θ/β	α/β	(α+θ)/β	(α+θ) /(α+β)	θ/β	α/β	(α+θ)/β	(α+θ) /(α+β)	Chance Level
1	−0.2194	0.7205	0.8198	−0.2191	−0.2599	−0.2295	−0.2678	−0.2386	0.3801	0.8416	0.8307	0.1819	−0.0021
2	0.1685	0.1368	0.1658	0.1615	0.1659	0.1819	0.1873	0.0074	−0.0262	0.3271	0.2836	−0.0219	0.0119
3	0.1231	0.5453	0.4642	0.1653	0.0882	−0.3300	−0.2355	0.4146	0.2453	0.6331	0.5728	0.2079	−0.0076
4	−0.0514	0.5348	0.6043	−0.0677	−0.0181	0.1585	0.0737	-0.1492	0.4086	0.7464	0.6881	0.2613	0.0134
5	0.2343	0.5685	0.6442	0.2299	0.2057	0.0357	0.0878	0.1525	−0.1410	0.6657	0.6531	−0.2695	0.0152
6	−0.3741	0.2702	0.2678	−0.3821	−0.4177	0.0113	−0.1646	−0.3937	−0.0634	0.7161	0.4527	−0.2018	0.0098
7	0.2609	−0.0211	−0.0047	0.2585	0.2463	0.0996	0.2252	0.2338	0.2696	0.7984	0.2950	0.2004	−0.0080
8	0.0596	0.1145	0.0328	0.0920	0.1413	−0.1028	−0.1336	0.2236	0.1160	0.2801	0.2050	0.1050	−0.0040
9	0.2983	0.5125	0.3660	0.3103	0.2308	0.0794	0.2078	0.2826	0.1933	−0.0178	−0.0458	0.2003	−0.0278
10	0.0656	0.7335	0.6305	0.0317	0.2022	0.4107	0.3902	−0.1374	0.1243	0.6850	0.6650	−0.0077	−0.0026
11	−0.2703	0.3677	−0.0631	−0.2700	−0.2449	−0.1866	−0.2472	−0.2368	−0.0511	0.2128	0.0357	−0.0575	0.0130
12	0.3299	0.1154	0.1080	0.3402	0.2705	0.0794	0.1813	0.2441	0.1456	0.3469	0.2843	0.0877	0.0082
13	0.2676	0.3305	0.3087	0.2620	0.2102	0.1310	0.1828	0.1468	−0.0967	0.3808	0.3579	−0.0816	0.0206
14	−0.1811	0.6256	0.4644	−0.1820	−0.2874	−0.1062	−0.2041	−0.2825	−0.0066	0.7065	0.5697	−0.1356	−0.0098
15	0.2884	0.3607	0.4678	0.2869	0.2278	0.2258	0.2315	0.2144	−0.0320	0.7359	0.5811	−0.2392	−0.0040
16	0.0665	0.2709	0.3736	0.0656	0.0379	0.0445	0.0421	0.0609	−0.0390	0.5515	0.4489	−0.1012	−0.0042
17	0.2016	0.2459	0.3907	0.1972	0.1547	0.1062	0.1376	0.0565	0.2444	0.7230	0.5474	0.2002	0.0062
18	−0.0400	0.4886	0.6983	−0.0539	−0.0377	0.0580	0.0028	−0.1255	0.5471	0.8021	0.7564	0.4321	0.0014
19	0.0429	0.8065	0.6613	0.0501	0.0281	−0.0608	−0.0333	0.0737	−0.0869	0.4373	0.4143	−0.1500	0.0086
20	−0.0192	0.6410	0.6480	−0.0147	−0.0272	−0.1442	−0.0373	−0.0039	0.3286	0.7135	0.6786	0.1714	0.0283
21	−0.3851	0.3108	0.1864	-0.3883	−0.2926	−0.2538	−0.2876	−0.2957	−0.0042	0.6313	0.4510	−0.0831	0.0186
22	−0.0359	−0.0943	−0.0130	−0.0279	−0.0336	−0.2058	−0.0841	0.0411	0.1228	0.4915	0.1917	0.0472	0.0038
23	0.2601	0.6180	0.4211	0.2560	0.1577	0.0099	0.0865	0.1321	0.1577	0.4714	0.4446	0.0587	0.0251
Mean	0.0474	0.4001	0.3758	0.0479	0.0325	0.0005	0.0148	0.0183	0.1190	**0.5600** **^†††^	0.4505	0.0319	0.0050

Significance: Wilcoxon signed-rank test across subjects. ** denotes ST-SODE vs. DE with p<0.01. ^†††^ denotes ST-SODE vs. PSD with p<0.001. The bold number indicates the highest correlation.

**Table 3:** Accuracy comparison of DE, PSD-SVM and ST-SODE on the Vigilance dataset.

Subject	DE			PSD-SVM			ST-SODE		
	5 s	10 s	20 s	5 s	10 s	20 s	5 s	10 s	20 s
1	44.17	33.33	53.33	71.67	70.00	80.00	82.50	88.33	90.00
2	60.00	65.00	73.33	82.50	80.00	86.67	73.33	70.83	66.67
3	85.00	93.33	93.33	84.17	85.00	83.33	77.50	81.67	83.33
4	58.33	70.00	73.33	60.00	100.00	86.67	95.83	100.00	100.00
5	77.50	88.33	80.00	59.17	90.00	83.33	81.67	81.67	83.33
6	67.50	75.00	73.33	65.00	81.67	66.67	89.17	93.33	93.33
7	50.00	50.00	50.00	59.17	76.67	93.33	95.83	96.67	100.00
8	88.33	86.67	70.00	88.33	81.67	76.67	78.33	76.67	80.00
Mean (%)	66.35	70.21	70.83	71.25	83.13	82.08	**87.29** **^†^	**92.29** **^††^	**93.75** **^††^

Significance: Wilcoxon signed-rank test across subjects. ** denotes ST-SODE vs. DE with *p* < 0.01; ^†^/^††^ denote ST-SODE vs. PSD-SVM with *p* < 0.05/0.01. The bold numbers indicate the highest accuracy for each setting.

**Table 4:** Accuracy comparison of DE-SVM (within-subject), PSD-SVM (within-subject), and ST-SODE (cross-subject) on the Vigilance dataset.

Subject	DE-SVM (Within-Subject)			PSD-SVM (Within-Subject)			ST-SODE		
	5 s	10 s	20 s	5 s	10 s	20 s	5 s	10 s	20 s
1	98.33	98.33	96.67	95.00	98.33	96.67	82.50	88.33	90.00
2	99.17	100.00	100.00	86.67	93.33	94.17	73.33	70.83	66.67
3	100.00	98.33	93.33	90.00	93.33	88.33	77.50	81.67	83.33
4	98.33	95.00	100.00	95.00	95.00	100.00	95.83	100.00	100.00
5	99.17	96.67	100.00	88.33	94.17	95.00	81.67	81.67	83.33
6	98.33	100.00	96.67	96.67	100.00	96.67	89.17	93.33	93.33
7	95.00	98.33	96.67	95.00	98.33	96.67	95.83	96.67	100.00
8	99.17	96.67	96.67	89.17	90.00	91.67	78.33	76.67	80.00
Mean (%)	98.44	97.92	97.50	92.08	95.83	95.83	87.29	92.29	93.75

**Table 5:** Average results of ST-SODE for all trials on the vigilance task and fatigue task using different aggregation window lengths (5 s, 10 s, 20 s).

Subject	Vigilance (1-Back)	Fatigue (2-Back)	Vigilance	Fatigue	Vigilance	Fatigue
1	0.000079	0.000893	0.000083	0.000868	0.000082	0.000993
2	0.000091	0.002179	0.000093	0.002197	0.000092	0.002282
3	0.000254	0.121783	0.000251	0.140796	0.000243	0.165035
4	0.000031	0.117506	0.000032	0.117510	0.000032	0.115746
5	0.000257	0.219803	0.000257	0.232860	0.000261	0.328026
6	0.000168	0.022431	0.000159	0.022737	0.000169	0.023496
7	0.000066	0.082160	0.000070	0.081650	0.000071	0.088062
8	0.000039	0.000862	0.000037	0.000926	0.000038	0.000937
Mean	0.000077	0.064361	0.000075	0.068261	0.000076	0.084273

## Data Availability

The SEED-VIG dataset is available on request at: https://bcmi.sjtu.edu.cn/home/seed/seed-vig.html (accessed on 5 April 2024). The Vigilance dataset presented in this study is available on request from the corresponding author. The data are not publicly available due to privacy restrictions.

## References

[1] World Health Organization (2024). Global Status Report on Road Safety 2023.

[2] Zhang H., Wu C., Yan X., Qiu T.Z. (2016). The effect of fatigue driving on car following behavior. Transp. Res. Part F Traffic Psychol. Behav..

[3] Zou S., Qiu T., Huang P., Bai X., Liu C. (2020). Constructing multi-scale entropy based on the empirical mode decomposition (EMD) and its application in recognizing driving fatigue. J. Neurosci. Methods.

[4] Fountas G., Pantangi S.S., Hulme K.F., Anastasopoulos P.C. (2019). The effects of driver fatigue, gender, and distracted driving on perceived and observed aggressive driving behavior: A correlated grouped random parameters bivariate probit approach. Anal. Methods Accid. Res..

[5] Chen J., Wang H., Wang Q., Hua C. (2019). Exploring the fatigue affecting electroencephalography based functional brain networks during real driving in young males. Neuropsychologia.

[6] Gu H., Ji Q. (2004). An automated face reader for fatigue detection. Proceedings of the Sixth IEEE International Conference on Automatic Face and Gesture Recognition.

[7] Rezaei M., Klette R. Look at the driver, look at the road: No distraction! no accident!. Proceedings of the IEEE Conference on Computer Vision and Pattern Recognition.

[8] Lal S.K., Craig A. (2001). A critical review of the psychophysiology of driver fatigue. Biol. Psychol..

[9] Bergasa L.M., Nuevo J., Sotelo M.A., Barea R., Lopez M.E. (2006). Real-time system for monitoring driver vigilance. IEEE Trans. Intell. Transp. Syst..

[10] Mårtensson H., Keelan O., Ahlström C. (2018). Driver sleepiness classification based on physiological data and driving performance from real road driving. IEEE Trans. Intell. Transp. Syst..

[11] Fan C., Peng Y., Peng S., Zhang H., Wu Y., Kwong S. (2021). Detection of train driver fatigue and distraction based on forehead EEG: A time-series ensemble learning method. IEEE Trans. Intell. Transp. Syst..

[12] Wang H., Xu L., Bezerianos A., Chen C., Zhang Z. (2020). Linking attention-based multiscale CNN with dynamical GCN for driving fatigue detection. IEEE Trans. Instrum. Meas..

[13] Alghanim M., Attar H., Rezaee K., Khosravi M., Solyman A., Kanan M.A. (2024). A hybrid deep neural network approach to recognize driving fatigue based on EEG signals. Int. J. Intell. Syst..

[14] Shi L.C., Jiao Y.Y., Lu B.L. (2013). Differential entropy feature for EEG-based vigilance estimation. Proceedings of the 2013 35th Annual International Conference of the IEEE Engineering in Medicine and Biology Society (EMBC).

[15] Youngworth R.N., Gallagher B.B., Stamper B.L. (2005). An overview of power spectral density (PSD) calculations. Opt. Manuf. Test. VI.

[16] Jap B.T., Lal S., Fischer P., Bekiaris E. (2009). Using EEG spectral components to assess algorithms for detecting fatigue. Expert Syst. Appl..

[17] Simonyan K., Zisserman A. (2015). Very deep convolutional networks for large-scale image recognition. Proceedings of the 3rd International Conference on Learning Representations (ICLR 2015).

[18] Deng P.Y., Qiu X.Y., Tang Z., Zhang W.M., Zhu L.M., Ren H., Zhou G.R., Sheng R.S. (2020). Detecting fatigue status of pilots based on deep learning network using EEG signals. IEEE Trans. Cogn. Dev. Syst..

[19] Hu F., Zhang L., Yang X., Zhang W.A. (2024). EEG-Based Driver Fatigue Detection Using Spatio-Temporal Fusion Network With Brain Region Partitioning Strategy. IEEE Trans. Intell. Transp. Syst..

[20] Du X., Meng Y., Qiu S., Lv Y., Liu Q. (2023). EEG emotion recognition by fusion of multi-scale features. Brain Sci..

[21] Buzsaki G., Draguhn A. (2004). Neuronal oscillations in cortical networks. Science.

[22] Klimesch W. (1999). EEG alpha and theta oscillations reflect cognitive and memory performance: A review and analysis. Brain Res. Rev..

[23] Borghini G., Astolfi L., Vecchiato G., Mattia D., Babiloni F. (2014). Measuring neurophysiological signals in aircraft pilots and car drivers for the assessment of mental workload, fatigue and drowsiness. Neurosci. Biobehav. Rev..

[24] Dinges D.F., Powell J.W. (1985). Microcomputer analyses of performance on a portable, simple visual RT task during sustained operations. Behav. Res. Methods Instrum. Comput..

[25] Duan R.N., Zhu J.Y., Lu B.L. (2013). Differential entropy feature for EEG-based emotion classification. Proceedings of the 2013 6th International IEEE/EMBS Conference on Neural Engineering (NER).

[26] Libert A., Van Hulle M.M. (2019). Predicting premature video skipping and viewer interest from EEG recordings. Entropy.

[27] Cui Y., Xu Y., Wu D. (2019). EEG-based driver drowsiness estimation using feature weighted episodic training. IEEE Trans. Neural Syst. Rehabil. Eng..

[28] Zhang C., Wang H., Fu R. (2013). Automated detection of driver fatigue based on entropy and complexity measures. IEEE Trans. Intell. Transp. Syst..

[29] Wang D., Tong J., Yang S., Chang Y., Du S. (2024). EEG Signal Driving Fatigue Detection based on Differential Entropy. Proceedings of the 2024 IEEE International Conference on Mechatronics and Automation (ICMA).

[30] Peng B., Zhang Y., Wang M., Chen J., Gao D. (2023). TA-MFFNet: Multi-feature fusion network for EEG analysis and driving fatigue detection based on time domain network and attention network. Comput. Biol. Chem..

[31] Shen K.Q., Li X.P., Ong C.J., Shao S.Y., Wilder Smith E.P. (2008). EEG-based mental fatigue measurement using multi-class support vector machines with confidence estimate. Clin. Neurophysiol..

[32] Li Y., Wang D., Liu F. (2022). The auto-correlation function aided sparse support matrix machine for EEG-based fatigue detection. IEEE Trans. Circuits Syst. II Express Briefs.

[33] Wang F., Wu S., Ping J., Xu Z., Chu H. (2021). EEG driving fatigue detection with PDC-based brain functional network. IEEE Sens. J..

[34] Zheng W., Lu B. (2017). A multimodal approach to estimating vigilance using EEG and forehead EOG. J. Neural Eng..

[35] Tuncer T., Dogan S., Ertam F., Subasi A. (2021). A dynamic center and multi threshold point based stable feature extraction network for driver fatigue detection utilizing EEG signals. Cogn. Neurodyn..

[36] Chai R., Naik G.R., Nguyen T.N., Ling S.H., Tran Y., Craig A., Nguyen H.T. (2016). Driver fatigue classification with independent component by entropy rate bound minimization analysis in an EEG-based system. IEEE J. Biomed. Health Inform..

[37] Pratticò D., Laganà F. (2025). Infrared Thermographic Signal Analysis of Bioactive Edible Oils Using CNNs for Quality Assessment. Signals.

[38] Al-Saegh A., Dawwd S.A., Abdul-Jabbar J.M. (2021). Deep learning for motor imagery EEG-based classification: A review. Biomed. Signal Process. Control.

[39] Li R., Wang L., Sourina O. (2022). Subject matching for cross-subject EEG-based recognition of driver states related to situation awareness. Methods.

[40] Wang K., Qiu S., Wei W., Yi W., He H., Xu M., Jung T., Ming D. (2023). Investigating EEG-based cross-session and cross-task vigilance estimation in BCI systems. J. Neural Eng..

[41] Barwick F., Arnett P., Slobounov S. (2012). EEG correlates of fatigue during administration of a neuropsychological test battery. Clin. Neurophysiol..

[42] Borghini G., Vecchiato G., Toppi J., Astolfi L., Maglione A., Isabella R., Caltagirone C., Kong W., Wei D., Zhou Z. (2012). Assessment of mental fatigue during car driving by using high resolution EEG activity and neurophysiologic indices. Proceedings of the 2012 Annual International Conference of the IEEE Engineering in Medicine and Biology Society.

[43] Craig A., Tran Y., Wijesuriya N., Middleton J. (2012). Fatigue and tiredness in people with spinal cord injury. J. Psychosom. Res..

[44] Lal S.K., Craig A., Boord P., Kirkup L., Nguyen H. (2003). Development of an algorithm for an EEG-based driver fatigue countermeasure. J. Saf. Res..

[45] Li G., Huang S., Xu W., Jiao W., Jiang Y., Gao Z., Zhang J. (2020). The impact of mental fatigue on brain activity: A comparative study both in resting state and task state using EEG. BMC Neurosci..

[46] Hayes M.H. (1996). Statistical Digital Signal Processing and Modeling.

[47] Kwak S.G., Kim J.H. (2017). Central limit theorem: The cornerstone of modern statistics. Korean J. Anesthesiol..

[48] Van der Vaart A.W. (2000). Asymptotic Statistics.

[49] Kirchner W.K. (1958). Age differences in short-term retention of rapidly changing information. J. Exp. Psychol..

[50] Tanaka M., Shigihara Y., Ishii A., Funakura M., Kanai E., Watanabe Y. (2012). Effect of mental fatigue on the central nervous system: An electroencephalography study. Behav. Brain Funct..

[51] Pergher V., Wittevrongel B., Tournoy J., Schoenmakers B., Van Hulle M.M. (2019). Mental workload of young and older adults gauged with ERPs and spectral power during N-Back task performance. Biol. Psychol..

[52] Chen K., Liu Z., Liu Q., Ai Q., Ma L. (2022). EEG-based mental fatigue detection using linear prediction cepstral coefficients and Riemann spatial covariance matrix. J. Neural Eng..

[53] Karim E., Pavel H.R., Jaiswal A., Zadeh M.Z., Theofanidis M., Wylie G., Makedon F. An EEG-based cognitive fatigue detection system. Proceedings of the 16th International Conference on Pervasive Technologies Related to Assistive Environments.

[54] van der Maaten L., Hinton G. (2008). Visualizing data using t-SNE. J. Mach. Learn. Res..

[55] Silverman D., Chen C., Chang S., Bui L., Zhang Y., Raghavan R., Jiang A., Le A., Darmohray D., Sima J. (2025). Activation of locus coeruleus noradrenergic neurons rapidly drives homeostatic sleep pressure. Sci. Adv..

[56] Osorio-Forero A., Foustoukos G., Cardis R., Cherrad N., Devenoges C., Fernandez L.M., Lüthi A. (2025). Infraslow noradrenergic locus coeruleus activity fluctuations are gatekeepers of the NREM–REM sleep cycle. Nat. Neurosci..

[57] Doran S.M., Van Dongen H., Dinges D.F. (2001). Sustained attention performance during sleep deprivation: Evidence of state instability. Arch. Ital. Biol..

[58] Dijk D.J., Czeisler C.A. (1995). Contribution of the circadian pacemaker and the sleep homeostat to sleep propensity, sleep structure, electroencephalographic slow waves, and sleep spindle activity in humans. J. Neurosci..

[59] Torsvall L. (1987). Sleepiness on the job: Continuously measured EEG changes in train drivers. Electroencephal. Clin. Neurophysiol..

[60] Van Dongen H.P., Maislin G., Mullington J.M., Dinges D.F. (2003). The cumulative cost of additional wakefulness: Dose-response effects on neurobehavioral functions and sleep physiology from chronic sleep restriction and total sleep deprivation. Sleep.

[61] Boksem M.A., Meijman T.F., Lorist M.M. (2005). Effects of mental fatigue on attention: An ERP study. Cogn. Brain Res..

[62] Liu Y., Xiang Z., Yan Z., Jin J., Shu L., Zhang L., Xu X. (2024). CEEMDAN fuzzy entropy based fatigue driving detection using single-channel EEG. Biomed. Signal Process. Control.

[63] Ojo J., Omilude L., Adeyemo I. (2017). Fatigue detection in drivers using eye-blink and yawning analysis. Int. J. Comput. Trends Technol..

[64] Xie Y., Chen K., Murphey Y.L. (2018). Real-time and robust driver yawning detection with deep neural networks. Proceedings of the 2018 IEEE Symposium Series on Computational Intelligence (SSCI).

[65] Gastaldi M., Rossi R., Gecchele G. (2014). Effects of driver task-related fatigue on driving performance. Procedia-Soc. Behav. Sci..

[66] Bibbò L., Angiulli G., Laganà F., Pratticò D., Cotroneo F., La Foresta F., Versaci M. (2025). MEMS and IoT in HAR: Effective Monitoring for the Health of Older People. Appl. Sci..

